# Potential New Avian Species as Carriers of Diverse Circoviruses

**DOI:** 10.3390/pathogens14060540

**Published:** 2025-05-28

**Authors:** Yasmin Luisa Neves Lemes Garcia, Ana Júlia Chaves Gomes, Guilherme Guerra Neto, Natasha Fujii Ando, Camila Sanches Rodrigues, Richard Alegria Cesario, Camila Domit, Fábio Henrique Lima, Helena Lage Ferreira, João Pessoa Araújo, Bruna Lindolfo da Silva, Fernando Rosado Spilki, Luciano Matsumiya Thomazelli, Thais Helena Martins Gamon, Isabela Barbosa Assis, Edison Luiz Durigon, Danielle Bruna Leal Oliveira, Vivaldo Gomes da Costa, Marília de Freitas Calmon, Paula Rahal

**Affiliations:** 1 Genomics Laboratory, Department of Biology, São Paulo State University (UNESP), São José do Rio Preto 15054-000, SP, Brazil; yasminlnlgarcia@gmail.com (Y.L.N.L.G.); aj.gomes@unesp.br (A.J.C.G.); 2 Zoobotanical Garden of São José do Rio Preto, São José do Rio Preto 15053-600, SP, Brazil; 3 Ecology and Conservation Laboratory, Federal University of Paraná (UFPR), Pontal do Paraná 83255-976, PR, Brazil; 4 Applied Preventive Veterinary Medicine Laboratory, Department of Veterinary Medicine, University of São Paulo (USP), Pirassununga 05508-220, SP, Brazil; 5 Veterinary Molecular Diagnostic Laboratory, Institute of Biotechnology, São Paulo State University (UNESP), Botucatu 01049-010, SP, Brazil; 6 Molecular Microbiology Laboratory, Feevale University, Novo Hamburgo 93525-075, RS, Brazil; 7 Laboratory of Clinical and Molecular Virology, Department of Microbiology, University of São Paulo, São Paulo 05508-220, SP, Brazil; 8 Albert Einstein Israelite Hospital, São Paulo 05652-900, SP, Brazil

**Keywords:** circovirus, birds, raven circovirus, Brazil, new host

## Abstract

Avian species pose risks for transmitting viruses, including avian circoviruses, that could be a threat for conservation and introduction into commercial flocks. This study investigated the presence of circovirus in 81 avian species from different regions of Brazil, including the northwest region of São Paulo and the coast of Paraná. Blood samples and oropharyngeal, cloacal, and other organ swabs were collected. The samples were extracted and screened using nested PCR for the replicase gene. In positive cases, the samples were sequenced. Regarding the results, a total of 1528 swab samples were collected from 601 birds, of which 24 (4%, 95% CI: 2.4–5.6) tested positive for various circovirus subtypes. Most positive birds (92%, 22/24) were from the northwest region of São Paulo, mainly from the city of São José do Rio Preto (54%, 12/22). The study also identified the presence of circovirus subtypes in avian families that were not previously described. Furthermore, the presence of raven circovirus in the blood sample of a *Nyctibius griseus* (potoo), suggests the possibility of a new carrier of the virus. Ultimately, the findings underscore the complexity of the viral ecology of avian circoviruses, highlight the necessity of enhancing future studies, and emphasize the need to support health assessment of wildlife, including marine birds.

## 1. Introduction

Avian species, with their vast geographic distribution, pose risks for the transmission of infectious diseases, especially considering their ability to migrate long distances and inhabit environments shared by other animals [1,2,3]. Avian circovirus (CV), which is transmitted among various bird species, can cause severe disease. This disease is characterized by symptoms such as anorexia, diarrhea, lethargy, dehydration, and feather disorders in various avian species. It is also associated with an invasion of the lymphoreticular system and immunodeficiency [4,5,6]. As a result, these viruses cause lymphoid depletion and immunosuppression, which increase the severity of secondary infections, resulting in mortality [7,8].

CVs have a circular single-stranded DNA (ssDNA) genome of 1.7 to 2.1 kb and are non-enveloped viruses with icosahedral T = 1 symmetry [9,10]. Their genomes are the smallest among viruses that infect animals, and the members of the family *Circoviridae* are differentiated by the position of the origin of replication related to the coding regions and the length of the intergenic regions, resulting in the classification into two different genera: *Cyclovirus* and *Circovirus* [11,12]. In particular, viruses belonging to the CV genus have an ambisense genome with similar genomic organization, including a stem–loop structure related to the initiation of rolling circle replication [13]. There are two conserved open reading frames: ORF V1 related to the coding of the replicase protein (Rep) and ORF C1, which encodes the capsid protein (Cap) [14].

Moreover, the genus CV consists of different viral species such as porcine circoviruses types 1, 2, and 3 (PCV1, PCV2 and PCV3). The PCV1 was identified as contaminants in pig kidney cell lines PK-15 and was the first demonstration of an animal virus with circular ssDNA [15]. Besides PCVs, different avian circoviruses have been reported over the years, such as *pissitacine beak and feather disease circovirus* (BFDV) [16], *pigeon circovirus* (PiCV) [17], *canary circovirus* (CaCV) [6,18], *goose circovirus* (GoCV) [19] and *raven circovirus* (RaCV) [20].

Brazil is considered one of the countries with the largest bird fauna, relevant for conservation in the Neotropical region, and is also the place with one of the largest numbers of endangered and endemic birds [21]. For this reason, efforts to conserve biodiversity are urgent and necessary, including diagnostics of viral diseases, such as those caused by circoviruses, which are important for assessing and monitoring the health and condition of the animal.

## 2. Materials and Methods

### 2.1. Ethics Statement

Animal procedures were approved by the Animal Care Committee of the University of São Paulo (USP), Pirassununga, Brazil (approval ID: 001686). Samples from the northwestern region of São Paulo were obtained from the Zoobotanical Garden of São José do Rio Preto, and the samples from the coast of Paraná were provided by the Ecology and Conservation Laboratory from the Center for Marine Studies (Federal University of Paraná/UFPR), as part of the Santos Basin Beach Monitoring Project (PMP-BS, “Projeto de Monitoramento de Praias da Bacia de Santos”). This project was required by the federal environmental agency in Brazil (IBAMA) for the environmental licensing of PETROBRAS.

### 2.2. Study Procedures

In the northwest region of São Paulo, samples were sent from the Zoobotanical Garden of São José do Rio Preto, renowned for its veterinary clinical treatment in around 110 nearby municipalities. Wild animals are rescued by environmental agencies and brought in for care when found in inappropriate or unusual situations, such as fires, road accidents, animal trafficking, and orphaned animals.

The samples from the coast of Paraná are part of a project to monitor beaches in the Santos Basin (PMP-BS). The project conducts daily monitoring along the Paraná coastline (approximately 90 km), collecting stranded animals, including those that are debilitated and carcasses. The animals were taken to the laboratory for veterinary care when found alive. In other cases, when they are dead, they are sent for necropsy to identify the causes of death and assess the health status of the animals, including birds, sea turtles, and marine mammals, using different diagnostic methods. Finally, the samples analyses were performed in the genomic studies laboratory (São Paulo State University/UNESP) located in São José do Rio Preto.

### 2.3. Samples from the Northwest Region of São Paulo

Between July 2022 and April 2024, swab samples were collected from birds from the oropharynx and cloaca, and when possible, blood samples were also collected, depending on the bird’s size and condition. The avian specimens were from 49 cities in the northwest region that arrived at the Zoobotanical Garden of São José do Rio Preto to receive clinical and emergency treatment. Most of the cities (*n* = 48) are located in São Paulo State, with one city in Minas Gerais. The collected samples were frozen (−80 °C) until analysis.

### 2.4. Samples from the Coast of Paraná

The samples from the state of Paraná were from December 2022 to August 2024. The Laboratory of Ecology and Conservation of the Marine Studies Center of the Federal University of Paraná (UFPR) collected oropharyngeal and cloaca swabs from avian species, as well as brain and pancreas samples. The last were collected from deceased animals. In total, the samples were from 6 coastal cities, sent to receive veterinary clinical treatment or, in the case of carcasses of dead animals, for necropsy. The samples were sent to be analyzed and were kept frozen at −80 °C during transport.

### 2.5. Total DNA Extraction

DNA from the samples was extracted using the UNIXTRACTOR DNA and RNA Extraction System (Uniscience, Osasco, Brazil). All solutions necessary for semi-automatic extraction were added to the 96-well Deepwell plate (Uniscience, Osasco, Brazil). Initially, a lysis solution composed of Guanidine Isocyanate (Merck Millipore^®^, Burlington, NJ, USA) and N-Lauroylsarcosine (Merck Millipore^®^, Burlington, NJ, USA)), magnetic Beads (Sera-Mag™ SpeedBeads), Proteinase K, and absolute Isopropanol (Merck Millipore^®^, Burlington, NJ, USA)) was prepared. The extracted material underwent washing steps using a mixture of DEPC-treated water and 80% ethanol (Sigma Aldrich^®^, Burlington, NJ, USA), and finally, the total extracted DNA was eluted in Tris-HCL.

### 2.6. Nested Polymerase Chain Reaction (nPCR)

The extracted material was supplied for used for nested PCR (nPCR) using the GoTaq^®^ Master Mix kit (Promega, Madison, WI, USA) and generic primers for the Replicase gene [22]. Cycling was performed in a Veriti Thermal Cycler (Applied Biosystems^®^, California, CA, USA). The first round of PCR included a 5 min incubation at 95 °C, followed by 45 cycles, each consisting of 94 °C for 30 s, 46 °C for 1 min, and 72 °C for 1 min, and a final incubation at 72 °C for 5 min. The second round was almost identical, except for the annealing temperature, which was 56 °C. Subsequent analysis of the PCR products was performed via electrophoresis in a 1.5% agarose gel using the Tris-base (TEB) solution, with visualization achieved through ethidium bromide staining. The controls were internal, and the marker used was the GeneRuler 1000 bp DNA Ladder (Thermo Scientific™, Waltham, MA, USA). Samples were considered positive when a 350 bp band was observed on the gel.

### 2.7. Sanger Sequencing

The samples were prepared using BigDyeTerminator v3.1 Cycle Sequencing Kit (Applied Biosystems^®^) following the manufacturer’s instructions and forward nested PCR primer (Cv-f2). The readings were performed using a Spectrum Compact CE System capillary sequencer (Promega). The sequences generated were analyzed using the GenBank^®^ tool in the Nucleotide Blast (BLASTn) program (https://blast.ncbi.nlm.nih.gov/Blast.cgi?PAGE_TYPE=BlastSearch (accessed on 6 February 2025)).

### 2.8. Isolation in Embryonic Eggs

All samples that tested positive for the circovirus replicase gene were subsequently submitted to the Laboratory of Clinical and Molecular Virology (BSL 3) Department of Microbiology from São Paulo University for viral isolation using standard methods, through inoculation in ten-day-old embryonated chicken eggs [23]. Briefly, at the BSL-3 Laboratory of the University of São Paulo, positive blood and cloacal swab samples from a potoo (*Nyctibius griseus*) were inoculated into embryonated chicken eggs to assess viral replication. Notably, only this individual tested positive in both the cloacal swab and blood samples, which may suggest that the presence of the virus in the bloodstream is indicative of active viral infectivity and highlights the potential of this species as a novel host. The samples were then centrifuged for 30 min at 3000× *g*, and the supernatant was then collected and passed through a membrane filter (0.22 μm). After this process, 0.2 mL was inoculated into the allantoic cavity of a 10-day-old specific pathogen-free (SPF) embryonated chicken egg and was incubated at 37 °C for 72–96 h. The allantoic fluid from the samples passed twice into the embryonated egg was then tested by nested PCR for viral detection.

### 2.9. Data Analysis

Excel 2016 (Microsoft Office 365) was used for data organization. Descriptive statistics were used to analyze categorical variables, which were expressed as frequencies and percentage of species and CV positivity in birds.

To determine the prevalence of CV in the collected samples, the sample size was determined using Thrusfield’s formula for an assumed infinite population [24]. The following parameters were applied: expected BFDV prevalence (30%), confidence interval (CI) 95%, and a *p*-value of 0.05 [25].

### 2.10. GenBank Accession Numbers

The GenBank accession numbers for sequences obtained by Sanger sequencing of positive samples amplified in nPCR for circovirus were PQ723684–PQ723708.

## 3. Results

### 3.1. Sample Characterization

**Figure 1:**
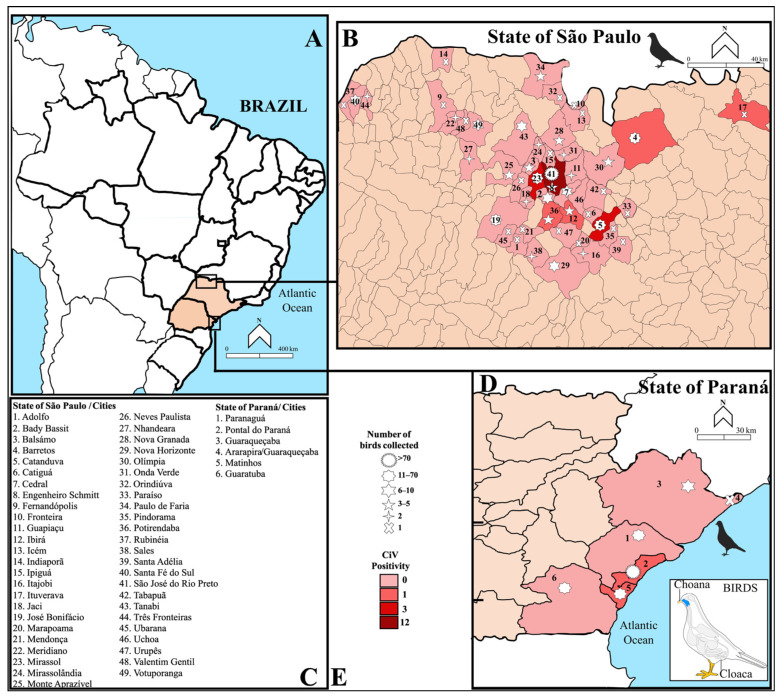
The geographical locations of the cities where the birds were collected are situated in the northwest region of São Paulo and the coast of Paraná, Brazil (**A**). (**B**) shows the sample cities in São Paulo, along with the number of samples collected and the number of birds positive for CV. (**C**) lists the 49 sampled cities in São Paulo and the six cities in Paraná. (**D**) presents the sampled cities in Paraná, along with the number of samples collected and the number of birds positive for CV. (**E**) displays symbols indicating the number of birds collected by geographic region, with a color bar representing the positivity rate for CV.

The birds obtained in this study represent a group of avifauna that occurs in the interior of São Paulo and the coast of Paraná (Figure 1). In the state of São Paulo, a total of 1064 samples (413 birds) were collected, including oropharyngeal swab (n = 413), cloacal swab (n = 413), and, when possible, due to the size and condition of the bird, blood (n = 238), covering 19 orders, 26 families, and identifying 59 species (Table 1). The most sampled species were *Psittacara leucophthalmus* (n = 83/413), *Patagioenas picazuro* (n = 24/413), *Eupsittula aurea* (n = 24/413), and *Ramphastos toco* (n = 24/413).

Regarding developmental stage, most birds were adults (306/413); however, samples were also obtained from juveniles (90/413) and chicks (17/413). Furthermore, of the 49 cities where the animals were rescued, the ones with the highest numbers of individuals analyzed were São José do Rio Preto (237/413), Catanduva (22/413), José Bonifácio (15/413), and Mirassol (13/413) (Figure 1A–C) (Table S1).

A total of 464 samples (188 birds) were analyzed from the coast of Paraná including swabs of oropharyngeal (n = 184), cloacal (n = 183), brain (n = 98), and pancreas (n = 1). In terms of sample diversity, there were five orders, 11 families, and 22 avian species. Unlike the samples from the northwest of São Paulo, the birds from the coast of Paraná were mostly from aquatic environments (Table 2).

The samples were from live animals (88/188), carcasses (94/188), and birds that arrived alive and later died (6/188). These specimens included adults (30/188), juveniles (78/188), chicks (3/188), and individuals with undetermined age classification (77/188). The most sampled cities of the state of Paraná were Pontal do Paraná (79/188), Paranaguá (40/188), and Guaratuba (32/188) (Figure 1A,C,D) (Table S2).

### 3.2. Molecular Detection of CV

Among the 601 birds examined—resulting in a total of 1528 samples—24 individuals tested positive, corresponding to a prevalence of 4% (95% CI: 2.4–5.6) (Figure 1E). A total of 34 samples collected from these 24 birds—10 of which yielded more than one positive sample—displayed CV positivity in nested PCR (Table 3). Of these, 29 produced high-quality sequences that could be reliably compared with the reference database, whereas the remaining five sequences were of insufficient quality to allow for viral identification. The majority of positive cases (22/24) were detected in birds from the northwestern region of São Paulo, while the remaining two were from the coastal region of Paraná. Regarding sample types, the highest detection rate was observed in cloacal swabs alone (11/24), followed by individuals in which both oropharyngeal and cloacal swabs tested positive (9/24), and those in which only oropharyngeal swabs were positive (3/24). In one individual (i.e., *Nyctibius griseus*), both the cloacal swab and blood tested positive (1/24).

Following sequencing using the Sanger method, 29 samples yielded sequences with identities corresponding to CV subtype. The six subtypes identified were *columbid circovirus* (CoCV) (9/29), *pigeon circovirus* (PiCV) (7/29), *beak and feather disease circovirus* (BFDV) (4/29), *porcine circovirus* 2 (PCV2) (3/29), *gull circovirus* (GuCV) (3/29), and *raven circovirus* (RaCV) (2/29). One sample was positive for a different viral genus belonging to the *Circoviridae* family, *Cyclovirus*. Additionally, eight birds of the 25 positive samples also had blood samples collected. After confirmation of the positive results, the blood samples were centrifuged to obtain the serum that underwent the same screening. Only one sample from *Nyctibius griseus* (potoo) was positive.

## 4. Discussion

In the analysis conducted, among the 81 avian species from the Northwest of São Paulo and the coast of Paraná, 12 species tested positive for CV subtypes, totaling 24 individuals. Cloacal swabs showed the highest proportion of positive results (11/24, 45%), followed by simultaneous positivity in both oropharyngeal and cloacal swabs (9/24, 37%), where both sample types yielded positive results concurrently. The majority of positive birds were adults (15/24; 62%), whereas juveniles accounted for 9/24 (37%). Most positive cases originated from the interior of São Paulo, particularly in São José do Rio Preto.

PiCV and CoCV are viral subtypes predominantly associated with infections in pigeon species, particularly *Columba livia domestica*, a member of the *Columbidae* family. Notably, one study showed a high frequency of PiCV in pigeons in southern Brazil (93.5%) [26,27,28,29]. Consistent with previous reports, the present study detected PiCV and CoCV in samples from birds of the *Columbidae* family, including four *Columba livia* and two *Patagioenas picazuro*.

On the other hand, the detection of PiCV- and CoCV-related DNA in *Caracara plancus*, *Coragyps atratus*, and *Cariama cristata*—species outside the *Columbidae* family—represents a novel and potentially significant observation. These results may indicate a broader host range for circoviruses than previously recognized. Although alternative explanations, such as passive ingestion or environmental contamination, must be considered, the presence of viral DNA in these species highlights the need for further investigation [27].

Similarly, the samples from individuals of the species *Coragyps atratus* that tested positive for PCV2 were from members of the *Cathartidae* family, which contrasts with the literature that associates this viral subtype with diseases and syndromes linked to infections in domestic and feral pigs [15,29]. Vultures, such as *Coragyps atratus*, are carnivorous, and therefore, the presence of the virus in these samples may be due to feeding on tissues, most likely from infected pigs. Other studies have demonstrated a correlation between the presence of viruses in vulture samples and the consumption of carcasses from infected animals [30,31,32].

BFDV, responsible for beak and feather disease in a variety of avian species belonging to the Psittacidae family, was previously identified in Brazil in studies involving *Cacatua alba*, *Amazona aestiva*, and *Ara ararauna* [25,33]. In the present study, two specimens of *Psittacara leucophthalmus* and one *Eupsittula aurea* tested positive for BFDV. In the study by Philadelpho et al. (2022) [25], a total of 41 out of 120 (34%) birds were positive for BFDV; however, these birds exhibited clinical signs consistent with CV infection. In contrast, the present study, which reported a lower overall positivity rate of 2 out of 601 (0.33%), involved randomly selected birds that did not exhibit any clinical signs of CV infection.

Circovirus-like infections have been identified in the black-backed gull (*Larus dominicanus),* as well as other gulls, since 1997, however, in the species *Sterna sp*., no case has been described [8,34,35,36]. Furthermore, a study reporting CV infection in a black-backed gull in New Zealand found that the infection resulted in lymphocyte depletion and immunosuppression, which allowed a very intense secondary fungal infection to develop [35].

The above subtypes are related to positive swab samples from the oropharyngeal and cloacal regions. Therefore, although some positive samples align with the findings in the literature, such as the cases of PiCV and CoCV in Columbidae and BFDV in psittacines, it is impossible to confirm whether the animals had CV infections. It is also necessary to consider that some bird species with predatory feeding habits (*Caracara plancus*, *Coragyps atratus* and *Cariama cristata*) may have come into contact with the virus during feeding, as well as other possible pathways that could explain the presence of the virus in the sample, which is not necessarily related to the susceptibility of the virus in a potential host [37].

The RaCV subtype, which is poorly characterized in the literature, was identified in two samples from the same avian specimen, a *Nyctibius griseus* (potoo). The blood and cloacal swab samples were collected from a bird that was referred for clinical and veterinary care at the Zoobotanical Garden of São José do Rio Preto. The presence of the virus in the blood may indicate an active infection process by RaCV, a hypothesis that was subsequently confirmed when the blood sample was isolated from embryonated eggs. Notably, the RaCV identified in the potoo sample illustrates the virus’s ability to infect and propagate, thus establishing a new host. These findings represent the first documented case of RaCV infection in *Nyctibius griseus*.

## 5. Conclusions

Based on the positive results of the study, a high prevalence of CV subtypes can be indicated in samples from avian species in the interior of São Paulo (22/601, 3.6%), as well as in samples from aquatic birds along the coast of Paraná (2/601, 0.33%). In particular, the highest positivity of samples was observed in cloacal swabs (11/24, 45%) and a greater infection rate in adult birds (15/24, 62% adults). The presence of CV subtypes in avian families that differ significantly from the range of hosts typically found in the literature may suggest new transmissions and even new hosts, as exemplified by the case of the potoo (*Nyctibius griseus*), identified as a new host of RaCV. The findings ultimately highlight the complex ecology of avian circoviruses, underscore the importance of improving future long-term studies, and stress the need to support wildlife health assessments, including for aquatic and seabirds.

## Figures and Tables

**Table 1:** Classification and abundance of avian species sampled in the northwest region of São Paulo.

Order	Family	*Scientific Name*	Number of Birds
-	-	*-*	1
Accipitriformes	Accipitridae	*Gampsonyx swainsonii*	2
		*Geranoaetus albicaudatus*	2
		*Harpia harpyja*	1
		*Ictinia plumbea*	1
		*Rupornis magnirostris*	19
Anseriformes	Anatidae	*Alopochen aegyptiaca*	1
		*Cairina moschata*	5
		*Dendrocygna autumnalis*	1
		*Nomonyx dominicus*	1
		*Spatula querquedula*	1
	Anhimidae	*Anhima cornuta*	1
Caprimulgiformes	Caprimulgidae	*Nyctidromus albicollis*	3
Cariamiformes	Cariamidae	*Cariama cristata*	22
Cathartiformes	Cathartidae	*Coragyps atratus*	19
Charadriiformes	Charadriidae	*Vanellus chilensis*	2
Columbiformes	Columbidae	*Columba livia*	10
		*Columbina*	2
		*Patagioenas picazuro*	26
		*Zenaida auriculata*	6
Coraciiformes	Momotidae	*Momotus momota*	1
Cuculiformes	Cuculidae	*Guira guira*	1
Falconiformes	Falconidae	*Caracara Plancus*	10
		*Falco sparverius*	5
		*Milvago chimachima*	1
Galináceos	Phasianidae	*Pavo cristatus*	1
Nyctibiiformes	Nyctibiidae	*Nyctibius griseus*	3
Passeriformes	Hirundinidae	*Pygochelidon* sp.	1
	Icteridae	*Gnorimopsar chopi*	1
		*Icterus pyrrhopterus*	1
	Thraupidae	*Sicalis flaveola*	1
		*Thraupis sayaca*	1
	Tyrannidae	*Pitangus sulphuratus*	4
		*Tyrannus melancholicus*	1
		*Tyrannus savana*	1
Pelecaniformes	Ardeidae	*Ardea alba*	1
		*Bubulcus-ibis*	3
		*Butorides striata*	1
		*Nycticorax nycticorax*	3
		*Syrigma sibilatrix*	2
	Threskiornithidae	*Mesembrinibis cayennensis*	1
		*Theristicus caudatus*	8
Piciformes	Picidae	*Colaptes campestres*	3
		*Melanerpes candidus*	1
	Ramphastidae	*Pteroglossus castanotis*	1
		*Pteroglossus* sp.	1
		*Ramphastos toco*	24
Psittaciformes	Psittacidae	*Amazona aestiva*	5
		*Amazona amazonica*	2
		*Ara ararauna*	13
		*Aratinga auricapillus*	3
		*Brotogeris chiriri*	20
		*Eupsittula aurea*	24
		*Psittacara leucophthalmus*	83
Strigiformes	Strigidae	*Athene cunicularia*	23
		*Megascops choliba*	3
		*Pulsatrix perspicillata*	1
		*Strix virgata*	5
	Tytonidae	*Tyto furcata*	21
Tinamiformes	Tinamidae	*Rhynchotus rufescens*	1
Trochiliformes	Trochilidae	*-*	1
Total			413

**Table 2:** Classification and abundance of avian species sampled in the coast of Paraná.

Order	Family	*Scientific Name*	Number of Birds
Charadriiformes	-	*-*	1
	Charadriidae	*Charadrius* sp.	1
		*Pluvialis dominica*	2
		*Pluvialis squatarola*	1
	Laridae	*Larus dominicanus*	38
		*Lorus dominicanus*	1
		*Rynchops niger*	3
		*Sterna hirundo*	2
		*Sterna* sp.	2
		*Thalasseus acuflavidus*	3
		*Thalasseus maximus*	2
	Scolopacidae	*Calidris canutus*	1
	Stercorariidae.	*Stercorarius* sp.	2
Pelecaniformes	Threskiornithidae	*Phimosus infuscatus*	3
Procellariiformes	Diomedeidae	*Thalassarche chlororhynchos*	1
	Procellariidae	*Calonectris* sp.	11
		*Daption capense*	1
		*Pterodroma* sp.	2
		*Puffinus puffinus*	11
Sphenisciformes	Spheniscidae	*Spheniscus magellanicus*	50
Suliformes	Fregatidae	*Fregata magnificens*	11
	Phalacrocoracidae	*Phalacrocorax brasilianus*	7
	Sulidae	*Sula leucogaster*	32
Total			188

**Table 3:** Positive avian species characterization and virus identity.

Region	*Species*	City	Positive Samples	Development Stage	Identity (%)	*Viral Subtype*
Northwest São Paulo state	*Caracara plancus*	São José do Rio Preto	Cloacal	Juveniles	98.02%	*columbid circovirus*
	*Caracara plancus*	Barretos	Cloacal	Juveniles	*n*	
	*Caracara plancus*	Mirassol	Cloacal	Juveniles	97.58%	*pigeon circovirus*
	*Coragyps atratus*	São José do Rio Preto	Oropharyngeal and cloacal	Juveniles	95.05%	*columbid circovirus*
	*Coragyps atratus*	São José do Rio Preto	Oropharyngeal and cloacal	Adults	99.58%	*porcine circovirus 2*
	*Coragyps atratus*	São José do Rio Preto	Cloacal	Adults	97.10%	*porcine circovirus 2*
	*Coragyps atratus*	Catanduva	Cloacal	Juveniles	99.09%	*columbid circovirus*
	*Columba livia*	São José do Rio Preto	Oropharyngeal and cloacal	Adults	93.18%	*pigeon circovirus*
	*Columba livia*	São José do Rio Preto	Oropharyngeal and cloacal	Adults	99.55%	*pigeon circovirus*
	*Columba livia*	São José do Rio Preto	Oropharyngeal and cloacal	Adults	99.52%	*columbid circovirus*
	*Columba livia*	Mirassol	Oropharyngeal	Adults	99.03%	*columbid circovirus*
	*Cariama cristata*	São José do Rio Preto	Cloacal	Adults	85.45%	*pigeon circovirus*
	*Eupsittula aurea*	Potirendaba	Oropharyngeal and cloacal	Adults	91.90%	*beak and feather disease virus*
	*Eupsittula aurea*	São José do Rio Preto	Oropharyngeal and cloacal	Adults	*n*	
	*Geranoaetus albicaudatus*	Ituverava	Cloacal	Juveniles	*n*	
	*Nyctibius griseus*	Catanduva	Cloacal and blood	Adults	81.30%	*raven circovirus*
	*Nyctibius griseus*	Catanduva	Cloacal	Adults	*n*	
	*Patagioenas picazuro*	São José do Rio Preto	Oropharyngeal	Juveniles	98.02%	*columbid circovirus*
	*Patagioenas picazuro*	São José do Rio Preto	Oropharyngeal and cloacal	Adults	98.00%	*columbid circovirus*
	*Psittacara leucophthalmus*	Mirassol	Oropharyngeal	Adults	90.90%	*beak and feather disease virus*
	*Psittacara leucophthalmus*	Ibirá	Cloacal	Adults	94.52%	*beak and feather disease virus*
	*Tyto furcata*	São José do Rio Preto	Cloacal	Adults	77.27%	*human associated cyclovirus 6*
Paraná Coast	*Sterna sp.*	Matinhos	Oropharyngeal and cloacal	Juveniles	83.30%	*gull circovirus*
	*Larus dominicanus*	Pontal do Paraná	Cloacal	Juveniles	85.60%	*gull circovirus*

*n* = no sequences.

## Data Availability

The original data presented in the study are openly available in UNESP Institutional Repository at [https://hdl.handle.net/11449/260663] (accessed on 6 February 2025).

## Supplementary Materials

The following supporting information can be downloaded at: https://www.mdpi.com/article/10.3390/pathogens14060540/s1. Table S1. Cities from which avian species were rescued and sent to the São José do Rio Preto Zoobotanical Garden to receive veterinary clinical treatment. Table S2. Cities from which avian species were rescued and sent to the Laboratory of Ecology and Conservation of the Marine Studies Center of the Federal University of Paraná (UFPR), to receive veterinary clinical treatment.

## Abbreviations

The following abbreviations are used in this manuscript:

BFDV	Pissitacine beak and feather disease circovirus
CaCV	Canary circovirus
Cap	Capsid protein
CV	Circovirus
GoCV	Goose circovirus
nPCR	Nested polymerase chain reaction
ORF	Open reading frame
PCV1	Porcine circovirus 1
PCV2	Porcine circovirus 2
PiCV	Pigeon circovirus
RaCV	Raven circovirus
Rep	Replicase protein
ssDNA	Single-stranded DNA
