# Supplementary material for: Potential New Avian Species as Carriers of Diverse Circoviruses
Source: Pathogens. 2025 May 28;14(6):540. doi: 10.3390/pathogens14060540 (PMC12196248; doi:10.3390/pathogens14060540)
Supplement: Supplementary file 1 [file pathogens-14-00540-s001.zip › pathogens-3592345-supplementary.pdf]

Supplementary Material

Table S1. Cities from which avian species were rescued and sent to the São José do Rio Preto Zoobotanical Garden to receive veterinary clinical treatment.

Cities	Number of birds collected
Adolfo	1
Bady Bassit	6
Balsámo	3
Barretos	11
Catanduva	22
Catiguá	1
Cedral	6
Engenheiro Schmitt	4
Fernandópolis	1
Fronteira*	1
Guapiaçu	2
Ibirá	5
Icem	1
Indiaporã	1
Ipiguá	1
Itajobi	2
Ituverava	1
Jaci	2
José Bonifácio	15
Marapoama	1
Mendonça	1
Meridiano	2
Mirassol	13
Mirassolândia	2
Monte Aprazível	3
Neves Paulista	1
Nhandeara	2
Nova Granada	4
Nova Horizonte	10
Olímpia	3
Onda Verde	2
Orindiúva	1
Paraíso	1
Paulo de Faria	4
Pindorama	1
Potirendaba	3
Rubinéia	1
Sales	2

Santa Adélia	1
Santa Fé do Sul	6
São José do Rio Preto	237
Tabapuã	1
Tanábi	7
Três Fronteiras	2
Ubarana	1
Uchoa	3
Urupês	1
Valentim Gentil	1
Votuporanga	8
Unidentified	2
Total	413

*City belonging to the state of Minas Gerais.

Table S2. *Cities from which avian species were rescued and sent to the Laboratory of Ecology and Conservation of the Marine Studies Center of the Federal University of Paraná (UFPR), to receive veterinary clinical treatment.*

**Region**	**Number of birds collected**
Paranaguá	40
Pontal do Paraná	79
Guaraqueçaba	7
Ararapira, Guaraqueçaba	1
Matinhos	27
Guaratuba	32
Total	188
